# Three Dimensional Membrane Vibration Measurement Using a Two Dimensional Position Sensitive Device

**DOI:** 10.3390/s23010174

**Published:** 2022-12-24

**Authors:** Rafał Białek, Kamila Białek

**Affiliations:** Faculty of Electronics, Military University of Technology, 00-908 Warsaw, Poland

**Keywords:** PSD, 3D measurements, vibration measurement

## Abstract

This study presents the outcome of research into membrane vibrations in a 3D space performed using a system based on position sensitive device (PSD) sensors. Here, measurements were conducted for harmonic vibrations. The use of such detectors for assessing the movement of objects within a plane or space requires determining the position of more than one marker. The article reviews two methods for detecting the position of several light spots on a sensor’s photosensitive plane: a marker sequential control method, and a method based on Fast Fourier Transform (FFT) that employs a square wave control signal. The authors present an approach to improving measurement accuracy for both methods. They also discuss the advantages and disadvantages of each.

## 1. Introduction

Position sensitive devices (PSDs) are commonly employed for noncontact measurement of distance and displacements. The sensors are characterized by high positioning resolution. PSD sensors are classified as sensors with an analogue output. A huge advantage of such sensors is their high speed of resolution. This is due to the wide bandwidth utilised (from DC up to even more than 150 kHz) [1,2,3,4,5,6]. Operation over a vast wavelength range (from approx. 400 nm to approx. 1050 nm) enables the sensor to be used in both visible and infrared light. Both one- and two-dimensional sensors are available on the market. The use of one marker enables the study of the motion of objects characterized by:

- one degree of freedom and moving in one direction, using a 1D PSD sensor,
- one or two degrees of freedom and moving over a plane, using a 2D PSD sensor.

When measuring 3D displacements, two-dimensional sensors are used. Often, however, the tested object is equipped with one active marker. A possible solution has been discussed in [7,8], which describes a measuring system with a single light source. Unfortunately, the use of one marker allows only the measurement of objects with three degrees of freedom. This paper presents a measuring system intended for recording the displacements of rigid bodies moving within a 3D space and with six degrees of freedom. The implementation of this task requires two bidimensional PSD sensors and three markers.

Preliminary studies on the methodology of determining the position of one or more light spots on photosensitive surfaces were published at the end of the 20th century. The authors of [9], for example, referred to a sequential control method and described a methodology that involves the application of pulse-amplitude modulation (PAM). It was concluded that measurement resolution, linearity, and accuracy are not affected by the use of multiple light sources. Moreover, the authors of [10] referred to the possibility of using a method based on code division multiple access (CDMA). In [11], the authors presented a Fast Fourier Transform (FFT) method which utilizes light intensity modulation with a harmonic function as a signal. The harmonic signal was also used in [12,13]. This type of approach is very important when processing PSD signals, due to their spectrum. Unfortunately, it is very burdensome, because it complicates the development of a marker controller. In addition, obtaining a low Total Harmonic Distortion (THD) coefficient is a major challenge. An analysis of the source literature did not provide any publications comparing selected methods.

The main objective of the present study was to analyse the capabilities exhibited by selected methods for detecting several light spots within a photosensitive plane of a PSD sensor, and to develop a measuring system that enables establishing object displacement within a 3D space. This research paper presents two methods that enable the position of more than one light spot on the photosensitive surface of a sensor to be determined. One method is based on sequential diode control, and the second is an FFT method built upon square wave signal. Square wave signal control is commonly employed in many systems. This is due to the fact that such a signal is very easy to generate using a microcontroller. This approach significantly facilitates the construction of a marker controller. This paper presents the results of 3D space membrane vibration measurements using a sequential diode control method, and describes the pros and cons of this measuring system. 

## 2. Materials and Methods

### 2.1. Light Spot Position Determination

**Figure 1:**
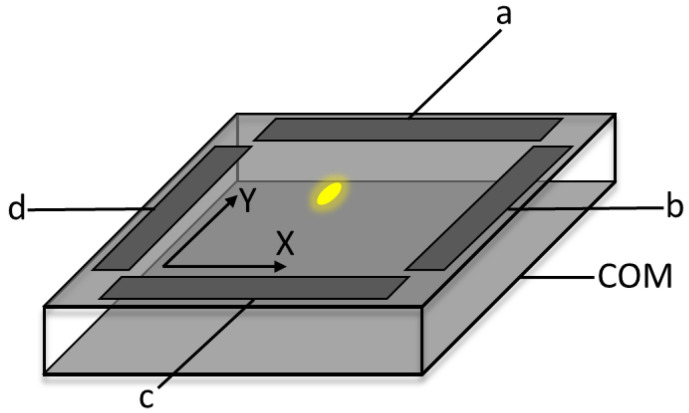
PSD structure.

Figure 1 shows an example of a PSD sensor structure.

The position of a light spot falling on a PSD 2D sensor plane is determined based on 4 current outputs of the sensor, in accordance with the formulas [14]:

(1) $$x=k_{x}\cdot \frac{I_{b}-I_{d}}{I_{b}+I_{d}}$$

(2) $$y=k_{y}\cdot \frac{I_{a}-I_{c}}{I_{a}+I_{c}}$$

where *k_x_* and *k_y_* are scaling coefficients that enable transformation to the Cartesian coordinate system and *I_a_*, *I_b_*, *I_c_* and *I_d_* are the currents in each electrode of the PSD sensor.

**Figure 2:**
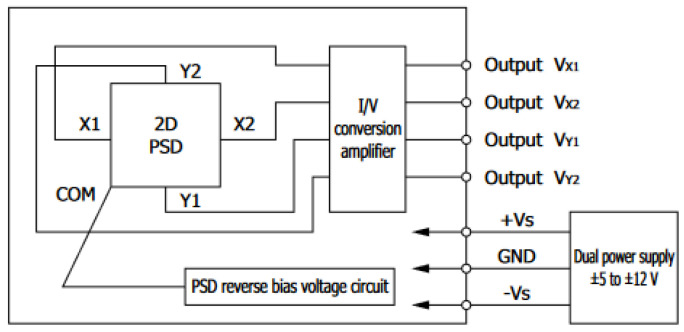
C10443-01 sensor block diagram [14].

The application of current outputs is very often inconvenient from the perspective of signal processing. Therefore, there are modules available on the market, the bodies of which contain both PSD and I/U converters, based on operational amplifiers. This research used two such modules. These were C10443-01 modules by Hamamatsu. Their block diagram is shown in Figure 2.

4 voltage outputs enable the *x* and *y* Cartesian coordinates to be ascertained according to the formulas [15]:

(3) $$x=\frac{\left(V_{X2}+V_{Y1}\right)-\left(V_{X1}+V_{Y2}\right)}{V_{X1}+V_{X2}+V_{Y1}+V_{Y2}}\cdot \frac{4.5}{2}$$

(4) $$y=\frac{\left(V_{X2}+V_{Y2}\right)-\left(V_{X1}+V_{Y1}\right)}{V_{X1}+V_{X2}+V_{Y1}+V_{Y2}}\cdot \frac{4.5}{2}$$

where *V_X_*_1_, *V_X_*_2_, *V_Y_*_1_ and *V_Y_*_2_ are the voltages of each of the four anodes of the PSD sensor.

Position determination using Equations (3) and (4) is often performed using analogue circuits based on operational amplifiers [14]. As a consequence, we obtain two voltage signals directly proportional to the x and y coordinates. Additionally, it is possible to determine the Σ parameter, which indicates incident light intensity [15].

(5) $$\sum =V_{X1}+V_{X2}+V_{Y1}+V_{Y2}$$

**Figure 3:**
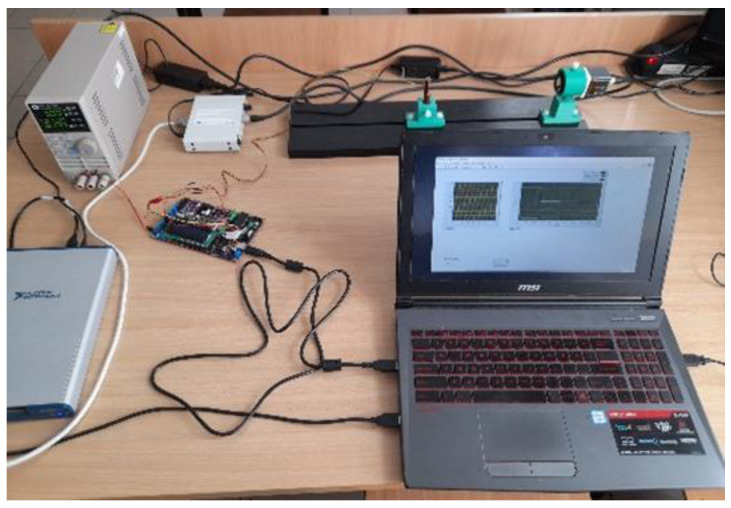
Image of the laboratory stand.

The market offers accessories that process signals from such modules using analogue technology. The authors employed two C10460 Hamamatsu processing units. Besides the *V_X_*_1_, *V_X_*_2_, *V_Y_*_1_, *V_Y_*_2_, *x*, *y*, ∑ analogue outputs, such units enable the transmission of data via an RS-232 interface. In addition, the device guarantees symmetrical power supply, which is required for the correct operation of the C10443-01 module. Only analogue signals were used for the purposes of this study. Data were acquired via a NI USB 6351 module and LabVIEW 2018 software provided by National Instruments. Numerical experiments were conducted in the MATLAB R2020b environment developed by MathWorks. Figure 3 shows the laboratory stand.

It should be stressed that it is impossible to determine the position of more than one light spot within a sensor plane when the markers operate in continuous wave mode (CW). Such an approach would result in the position being determined as the resultant of the position of the 3 spots [16]. Therefore, in order to detect at least two markers, it is necessary to develop methods based on the intermittent operation of radiation sources.

### 2.2. Methods for Determining the Position of Several Markers

**Figure 4:**
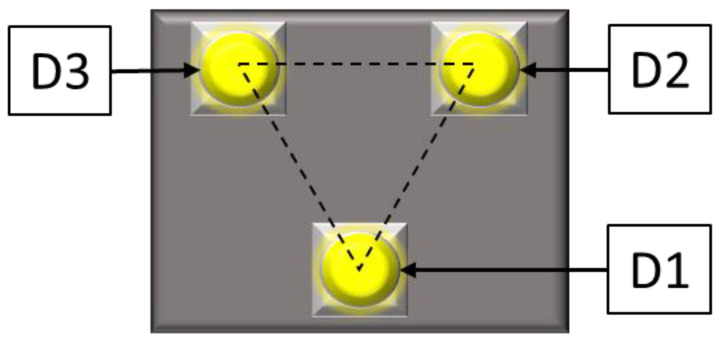
Markers.

This study only presents information pertaining to methods that can be employed when there is no communication between the marker control system and other elements of the measuring system. Figure 4 shows a model of a plate with three LED markers. The markers were installed on the vertices of an equilateral triangle with 10 mm sides. 

This study discusses two methods that make it possible to ascertain the position of more than one marker. These are the:

- Sequential marker control method;
- FFT-based method.

**Figure 5:**
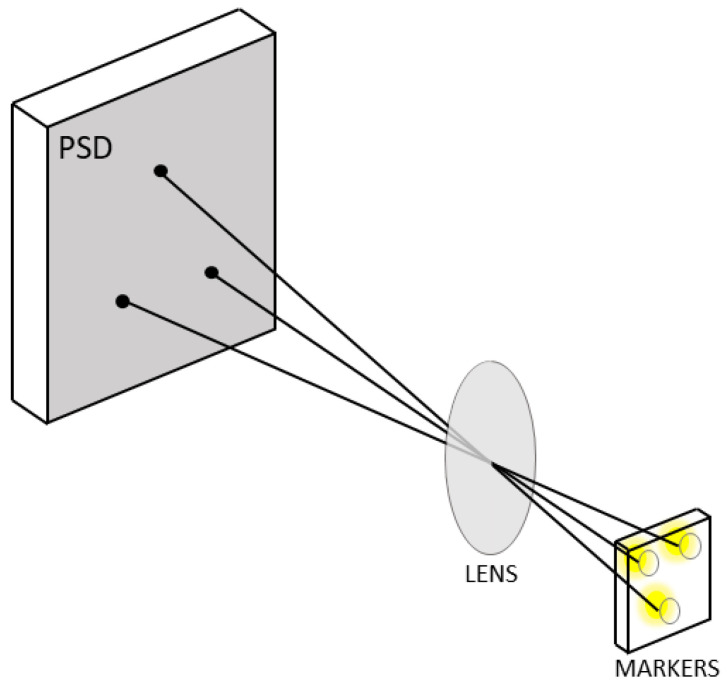
Projection.

Based on the position of light spots on the photosensitive plane of the sensor and the distance between markers, these methods enable marker positions in space to be determined (Figure 5). However, it should be emphasized that such a marked plate should be placed parallel to the PSD photosensitive plane.

#### 2.2.1. Sequential Marker Control Method

This method is based on controlling diodes in a strictly defined order. The following sequence was employed in this study:

- D1 diode activation;
- D2 diode activation;
- D3 diode activation;
- Deactivation of all diodes.

**Figure 6:**
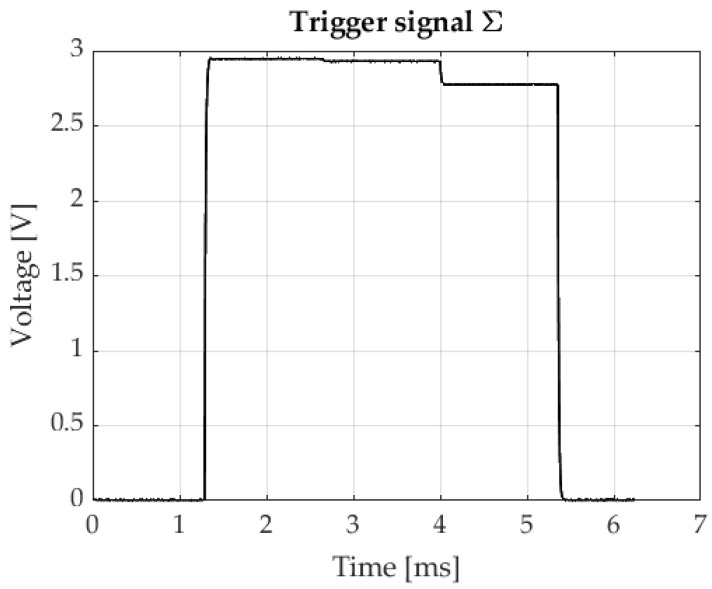
Trigger signal example.

In order to correctly ascertain the position of three light spots on the photosensitive surface of the sensor, it is necessary to define voltage values for sensor outputs at appropriate moments in time. Therefore, it is crucial to determine trigger conditions. These conditions can be defined in the voltage, time, and frequency domains. Σ, the value of which depends on what incident light intensity was applied, in this method serves as the trigger signal. An example waveform of this signal is shown in Figure 6.

**Figure 7:**
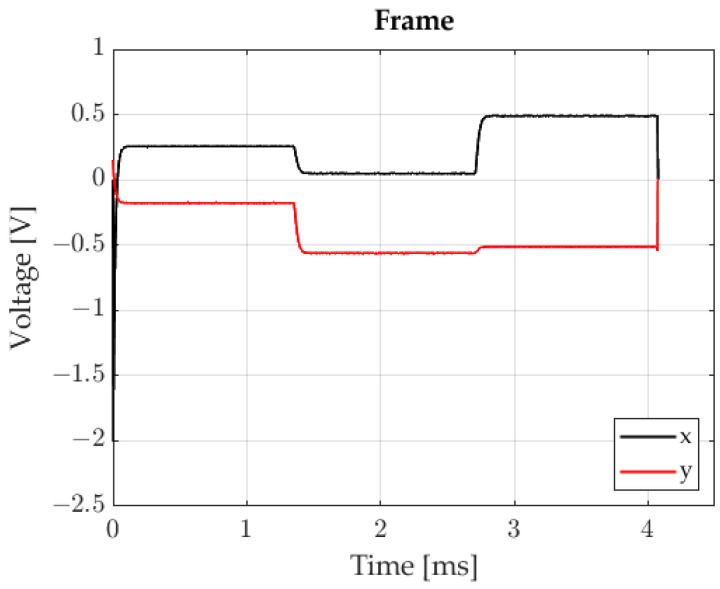
X and Y waveforms.

In this study, the process is triggered on the rising edge, upon the signal reaching a value of 1 V. Employing IR diodes of the same type is a key issue in the sequential marker control method. It is also important to ensure the possibility of regulation of the current flowing through individual diodes, because this influences the value of Σ. The trigger signal enables position readings to be taken at appropriate moments, when the relevant diodes are operating. Examples of x and y signals are shown in Figure 7. 

**Figure 8:**
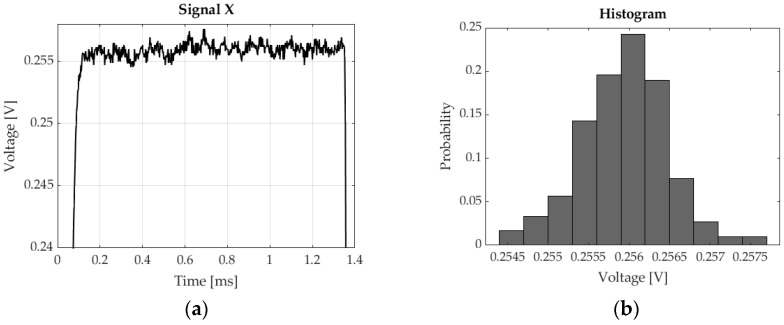
(**a**) Fragment of the x signal with visible noise; (**b**) noise histogram.

In this paper, x and y signals consist of 1636 samples. The signals were recorded with a sampling frequency of 400 kHz. The easiest way to assess the position of 3 light spots on the photosensitive surface of the sensor is to determine the x and y for specific moments in time, e.g., for 1.0 ms, 2.5 ms and 3.5 ms. Unfortunately, these signals are subject to noise, which adversely impacts position measurement accuracy (Figure 8a). Based on the samples within the time interval from 0.2 ms to 1.3 ms, it was possible to plot a histogram in order to establish the character of the unwanted signal (Figure 8b). 

**Figure 9:**
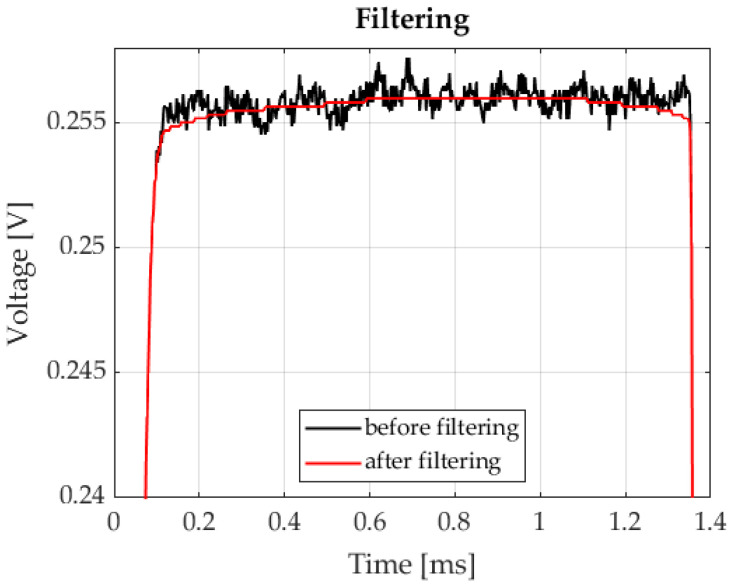
Signal after filtering.

Very similar histograms were obtained for many recorded signals and intervals corresponding to individual markers. Based on the obtained numerical experiment results, it was assumed that the interfering signal was normally distributed noise. The problem of Gaussian white noise in PSD sensors has been described in [17]. Studies have shown that the presence of this noise was visible throughout the entire band of the analysed signal. Therefore, applying classic digital filters will disable the separation of the information signal from noise. The best results were obtained after using a median filter. This is a nonlinear filter that is designed to reduce Gaussian noise and noise that often occurs in images, such as salt-and-pepper noise [18,19,20]. The authors of this study applied a median filter with a length of 501. Such odd-length filters are commonly employed in practice, because calculating the median does not involve determining an average value. The view of the post-filtration signal is shown in Figure 9. 

**Figure 10:**
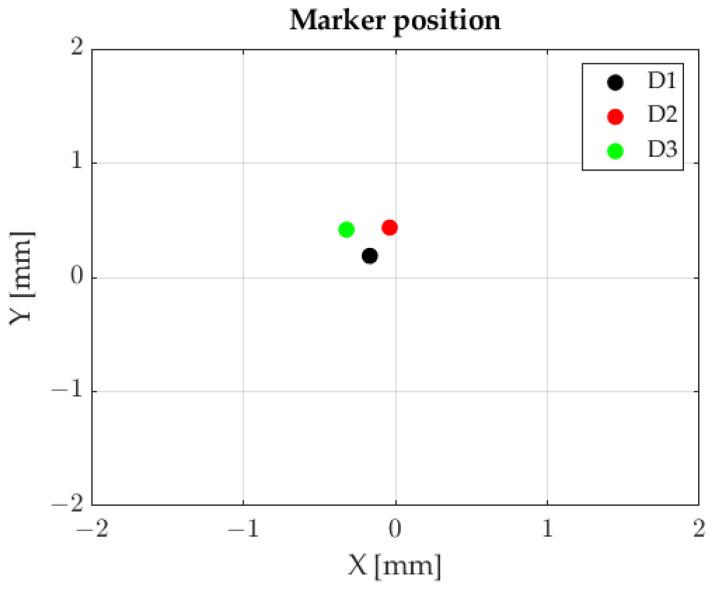
Markers’ positions, sequential marker control method.

Filtration was followed by the determination of the positions of light spots falling on the photosensitive plane of the sensor. The resultant view is shown in Figure 10. 

The constructed system enables the recording of position with a frequency of 100 Hz. Measurements at rest were conducted afterwards. 10,000 samples were recorded. Standard deviation and peak-to-peak value were established on this basis. The measurements were taken without and with a median filter. The results are shown in Table 1 and Table 2.

The conducted measurements indicate that the application of a median filter allowed a significant reduction in both standard deviation and peak-to-peak value. It should be added that a voltage equal to 1 V corresponds to 1 mm on the photosensitive plane of a PSD sensor.

#### 2.2.2. FFT-Based Method

This method involves diodes controlled by square wave signals with different frequencies and a duty factor equal to 50%. The spectrum of such control signals consists of odd harmonics with amplitudes consistent with the formula:

(6) $$A_{n}=\frac{4A}{n\pi }\;\;\;\;\;\;\;\;\;\;\;\;\;\;\;\;\;\;\;\;\;\;\;n=1,\;3,\;5,\;\ldots$$

where *A* is the square wave amplitude and n is the harmonic number.

First, the value of *f*_1_ frequency for a selected marker should be adopted. Next, the frequencies of the remaining signals should be matched, so that they fall in the range from *f*_1_ to 3*f*_1_, while being arranged as evenly as possible. 

The appropriate selection of sampling frequency and the number of samples are very important from the perspective of the digital processing of such signals. These parameters directly impact the frequency resolution according to the formula:

(7) $$\Delta f=\frac{f_{p}}{N}$$

where *f_p_* is the sampling frequency and *N* is the number of samples.

The frequencies of marker control signals should be multiples of Δ*f*. The measurement window should cover at least several periods of the signal with the lowest frequency. Windowing should be applied in order to minimize the leakage phenomenon. According to the theory of sampling, sampling frequency should be at least 2 times higher than the highest frequency within the spectrum of analysed signals. This approach prevents the phenomenon of aliasing. Unfortunately, the spectrum of ideal square wave signals consists of an infinite number of harmonics, making it impossible to satisfy this condition. However, according to Formula 6, the amplitudes of subsequent harmonics decrease with increasing n. Furthermore, there are no signals with infinitely steep edges in reality. However, it should be stressed that the upper frequency of the band in the PSD sensors employed is 16 kHz. Therefore, this element acts as an antialiasing filter. The experimental tests we conducted indicated that noise dominates in the spectrum of the analysed signal above a frequency of 120 kHz. 

Three markers were used in this study. They are controlled as follows:

- The D1 marker is controlled by a square wave signal with a frequency of 1.0 kHz;
- The D2 marker is controlled by a square wave signal with a frequency of 1.7 kHz;
- The D3 marker is controlled by a square wave signal with a frequency of 2.4 kHz.

**Figure 11:**
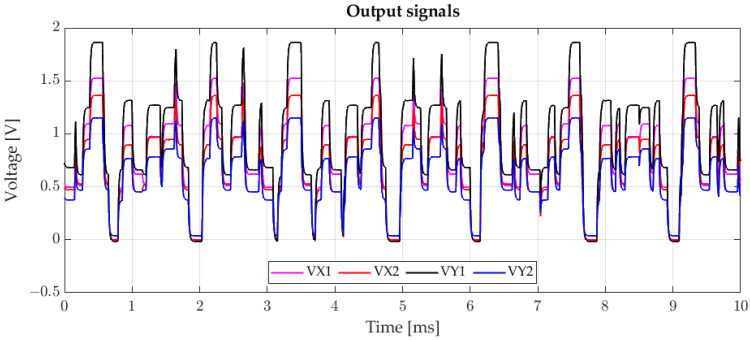
Output signals from PSD sensor.

Sampling frequency within this method was 250 kHz, and spectrum calculations were conducted for 2500 samples. These parameters enabled a frequency resolution of 100 Hz to be obtained. An example of signal time form is shown in Figure 11.

**Figure 12:**
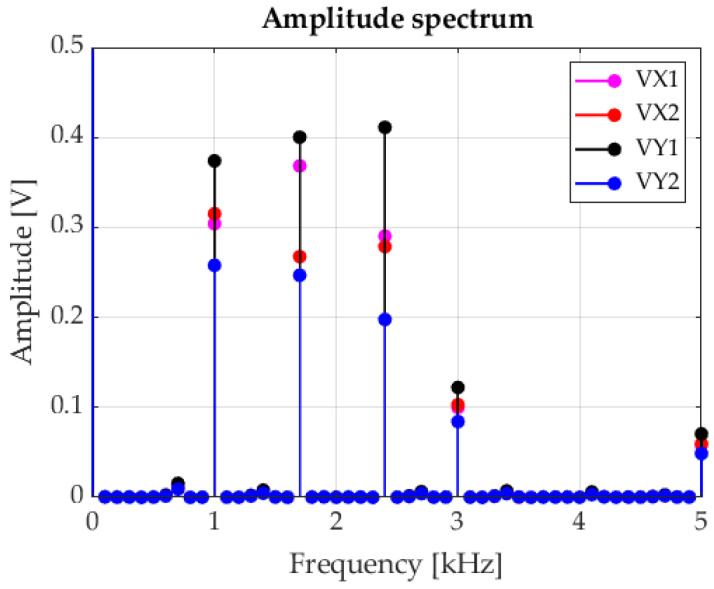
Amplitude spectrum.

The signals presented in Figure 11 were used as the foundation for establishing the amplitude spectrum using the FFT algorithm. The selected fragment is shown in Figure 12.

It should be emphasized that the position of individual markers is ascertained solely based on the amplitude spectrum, and is specified only on the *V_X_*_1_, *V_X_*_2_, *V_Y_*_1,_ and *V_Y_*_2_ component amplitudes occurring for the frequencies of: 1 kHz, 1.7 kHz, and 2.4 kHz. A great advantage of this method is that signal shift does not impact the amplitude characteristics. Instead, this only leads to changes in the phase characteristics (which are negligible in this method). As a result, selecting the trigger moment does not affect the result of determining the position of individual markers. Another advantage of applying the FFT method is the relatively low sensitivity of noise to the value of calculated useful signals’ spectral parameters (although, naturally, noise is visible throughout the entire analysed band). 

**Figure 13:**
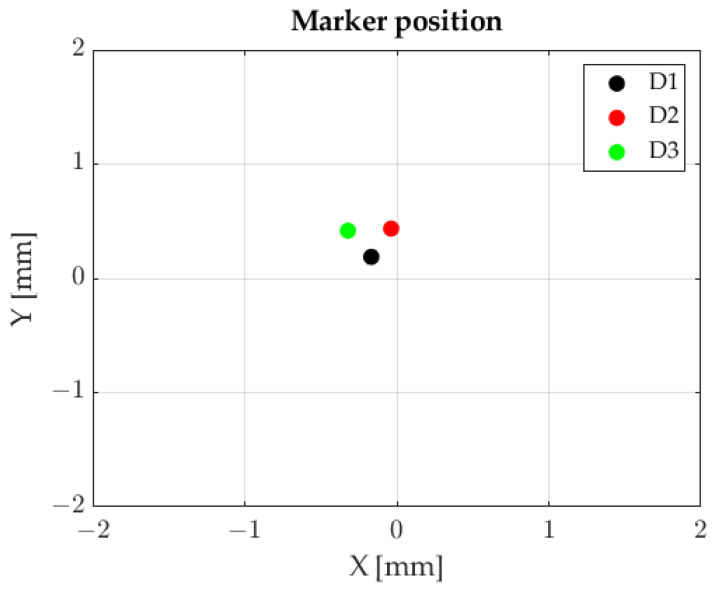
Markers’ positions, FFT method.

Employing FFT and Formulas 3 and 4 enabled us to determine the position of light spots falling on the photosensitive plane of the sensor. The results are shown in Figure 13. 

The constructed system enables position recording with a frequency of 100 Hz. Measurements at rest were conducted afterwards. 10,000 samples were recorded. Standard deviations and peak-to-peak values were established on this basis. Measurements were taken for the selected windowing functions. The results are shown in Table 3, Table 4, Table 5, Table 6 and Table 7.

The measurements conducted indicate that the application of the appropriate windowing functions enabled us to reduce the standard deviation and peak-to-peak values. The best results were obtained for the Hanning and Blackman windows. 

Moreover, the conducted measurements indicate that both the sequential marker control method and FFT method can be successfully employed.

## 3. Results

**Figure 14:**
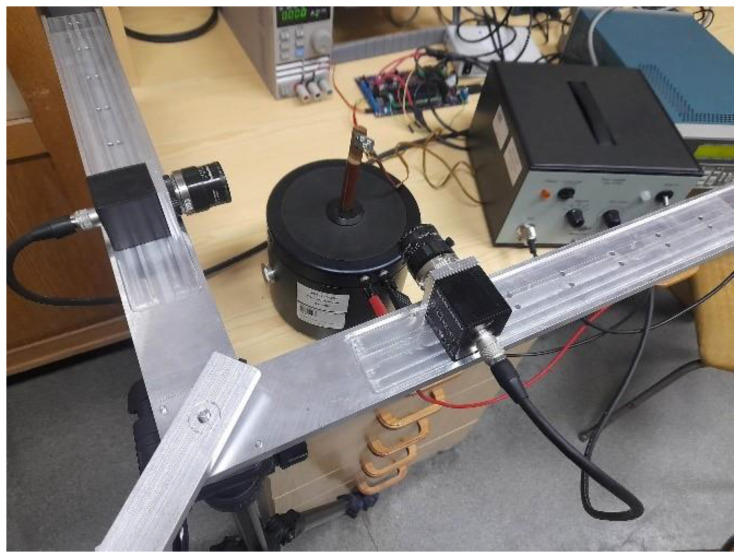
Laboratory stand for vibration measurements.

This section presents the results of 3D measurements of membrane vibrations using the sequential marker control method. Implementing this task required employing two 2D PSD sensors. In this study, the sensors were placed orthogonally relative to each other. Vibrations were induced using the 4809 vibration exciter by Brüel & Kjær. A view of the measuring stand is shown in Figure 14.

**Figure 15:**
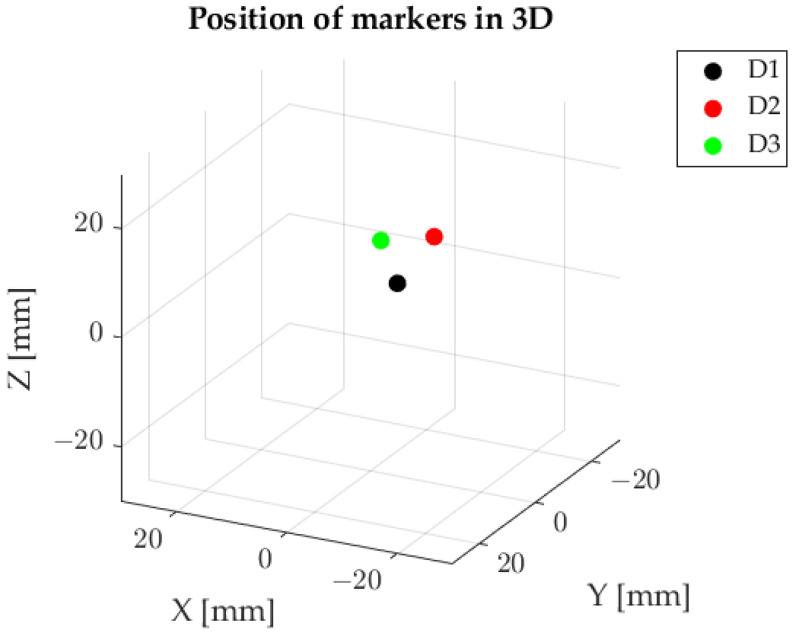
Markers’ positions in 3D.

Markers’ positions within the Cartesian coordinate system are shown in Figure 15. Each of the markers is described by 3 coordinates (X, Y and Z).

**Figure 16:**
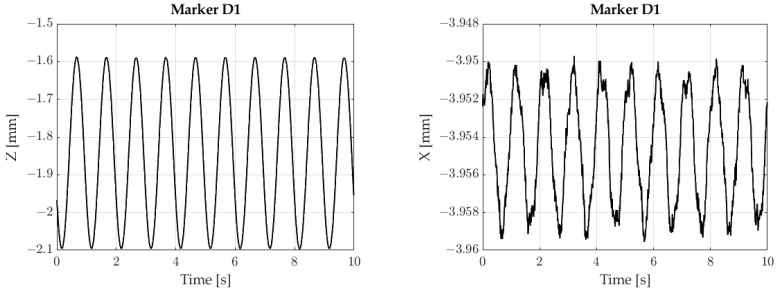
Recorded signals.

The next stage involved measuring membrane vibrations. Harmonic vibrations with an amplitude of 0.25 mm and a frequency of 1 Hz were studied. Figure 16 shows vibrations in the Z and X axes for the D1 marker.

**Figure 17:**
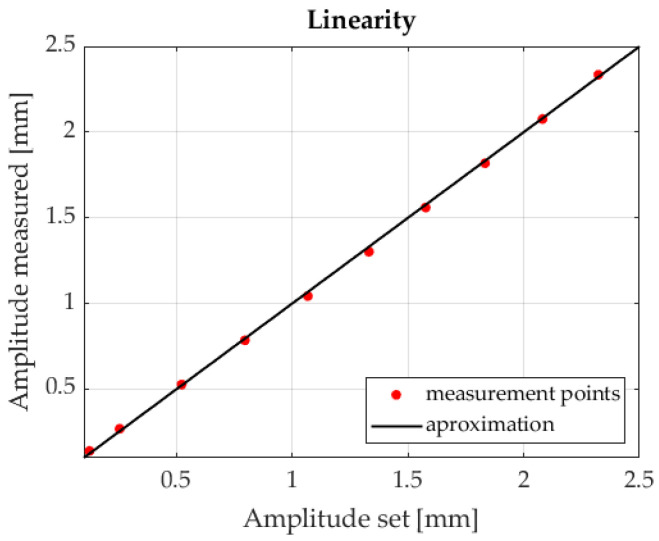
Vibration amplitude measurements.

Linearity measurements were presented in the last stage. Figure 17 reveals the dependence between the measured amplitude value and a preset value. A Pearson coefficient equal to 0.9998 was obtained for the resultant data.

## 4. Discussion

Vibrations can be measured in various ways. The most popular method of measurement uses accelerometers. A signal from this type of sensor is directly proportional to acceleration. Unfortunately, the determination of displacement requires double integration. This causes drift, which significantly limits measurement accuracy [21]. Another popular method of vibration measurement involves the use of cameras. Regrettably, this approach also has numerous restrictions. In this case, measurement resolution depends on the number of matrix pixels. For example, the authors of [22] present a study of speaker membrane vibrations using a Beasler acA2000-340 kc high-speed camera. It enables measurements to be conducted at a rate of 340 fps. Such a value is unacceptable in many situations. 

Employing a measuring system based on PSD sensors could turn out to be an attractive solution in many fields of application. Such devices have analogue outputs and are characterized by a very wide band. The main issue associated with this system is detecting the position of more than one light spot on the photosensitive plane of the sensor. The tests conducted as part of the research indicated that this detection could be executed using a sequential marker control system or a method based on FFT. Table 8 lists the advantages and disadvantages of both methods. 

The results presented in the paper indicate that it is possible to determine the positions of light spots within the photosensitive plane of a sensor. The potential determination of the positions of several light spots within a sensor’s photosensitive plane enables the construction of a measuring system intended for measurements within a 3D space. The system allows displacement measurements to be precisely conducted while maintaining linearity. The fundamental downside of the presented measuring system is the need to ensure visibility between the radiation source (marker) and receivers (PSD sensors). Unfortunately, this requirement makes it impossible to employ this measuring system in many applications. The disadvantages can also include the need to use active markers that must be attached to the tested object. Furthermore, a certain limitation is the fact that marker position is determined based on a particular signal fragment. This restricts the rate of position detection. Another issue is the possible occurrence of interference in useful signals. The PSD sensor employed in the study operates over a very wide wavelength range, from 320 nm to 1100 nm. The occurrence of additional radiation sources can distort system operation. For this reason, an extra bandpass optical filter adapted to the radiation emitted by the markers was used in the measuring system. 

The authors believe that the greatest advantage of the conducted tests is that they demonstrate the possibility of accurately determining the positions of three markers in a 3D space. The direction of further research is associated with conducting tests to assess measurement accuracy.

## 5. Conclusions

The paper presents an analysis of the capabilities exhibited by two selected methods for detecting several light spots on a photosensitive plane of a PSD sensor. The authors first reviewed current methods of improving measurement accuracy. The conducted tests indicate that both the sequential marker control method and FFT method can be successfully employed for detecting the positions of several light spots. In accordance with our findings, employing a median filter within the marker sequential control method significantly limited noise in the signal, thus reducing V_pp_ and Std values. With regard to the FFT-based method, the correct selection of the windowing function is extremely important. The best results were obtained using the Hanning and Blackman windows. Moreover, the conducted tests indicated that a square wave control signal can be successfully used as part of the method based on FFT. As a result, the authors presented a measuring system consisting of two PSD systems, intended to measure displacements within a 3D space. The constructed measuring system is versatile, enables measuring the movements of objects with six degrees of freedom, and allows effective recording of membrane harmonic vibrations. A strong linearity between the preset and measured amplitudes was obtained. The Pearson correlation coefficient was 0.9998.

## Figures and Tables

**Table 1:** Results without median filter.

	X1	X2	X3	Y1	Y2	Y3
**Std [uV]**	497.8	466.2	529.4	477.6	407.5	434.3
**V_pp_ [mV]**	3.863	3.541	4.024	3.702	3.541	3.058

**Table 2:** Results with median filter.

	X1	X2	X3	Y1	Y2	Y3
**Std [uV]**	78.0	66.7	84.2	81.3	79.3	63.3
**V_pp_ [mV]**	0.4829	0.4829	0.4829	0.4829	0.3219	0.3219

**Table 3:** Rectangular window results.

	X1	X2	X3	Y1	Y2	Y3
**Std [uV]**	832.3	871.4	512.0	578.9	928.3	808.9
**V_pp_ [mV]**	10.3	3.6	5.8	18.3	7.4	13.6

**Table 4:** Hanning window results.

	X1	X2	X3	Y1	Y2	Y3
**Std [uV]**	47.6	132.1	47.4	127.2	168.0	153.5
**V_pp_ [mV]**	0.3713	0.7019	0.4102	0.6287	0.9243	0.9227

**Table 5:** Blackman window results.

	X1	X2	X3	Y1	Y2	Y3
**Std [uV]**	61.4	140.5	44.6	78.6	125.0	124.8
**V_pp_ [mV]**	0.398	0.758	0.315	0.542	0.779	0.720

**Table 6:** Hamming window results.

	X1	X2	X3	Y1	Y2	Y3
**Std [uV]**	122.8	157.5	75.2	128.1	174.7	165.1
**V_pp_ [mV]**	2.5	0.9	0.8	6.5	3.5	2.3

**Table 7:** Flat top window results.

	X1	X2	X3	Y1	Y2	Y3
**Std [uV]**	1111.1	1065.9	196.3	657.7	497.1	1025.9
**V_pp_ [mV]**	4.1	4.0	1.1	2.5	2.3	4.3

**Table 8:** Market control method comparison.

	Sequential Method	Method Based on FFT
**Advantages**	-Square wave control signals -Does not require high stability of marker control signal frequency	-Compatible with a square wave control signal -Does not require a trigger signal
**Disadvantages**	-Requires use of a filter to improve measurement accuracy -Trigger signal is required	-Requires use of a windowing function to improve measurement accuracy -Requires high stability of marker control signal frequency

## Data Availability

The data presented in this study are available on request from the corresponding author.
